# MicroRNA response in insect salivary glands to plant virus infection

**DOI:** 10.1128/jvi.01434-25

**Published:** 2025-10-29

**Authors:** Yan Xiao, Guohua Liang, Jiaming Zhu, Feng Cui, Wan Zhao

**Affiliations:** 1 State Key Laboratory of Animal Biodiversity Conservation and Integrated Pest Management, Institute of Zoology, Chinese Academy of Sciences 53024 , Beijing, China; 2 University of Chinese Academy of Sciences, Beijing, China; Tsinghua University, Beijing, China

**Keywords:** plant virus, rice stripe virus, salivary glands, microRNA, small brown planthopper, viral transmission

## Abstract

Most arboviruses rely on insect vectors for transmission, with salivary glands serving as a critical gateway for viral spread to new hosts. MicroRNAs (miRNAs) are key regulators of gene expression, yet their roles in salivary glands during virus infection remain poorly understood. Using the small brown planthopper (SBPH)–rice stripe virus (RSV) system, we sequenced small RNAs from salivary glands of nonviruliferous and viruliferous insects and identified 5,909 known miRNAs from 201 families. Of these, 1,143 miRNAs were differentially expressed upon RSV infection, including 1,090 upregulated from 36 families and 53 downregulated from 11 families. These differentially expressed miRNAs were predicted to target 2,876 genes. Gene Ontology analysis showed that targets of upregulated miRNA were enriched in “protein binding,” while those of downregulated miRNAs were associated with “cytoskeleton” and “regulation of dephosphorylation.” The neurotrophin signaling pathway was the top-enriched KEGG pathway for the upregulated miRNA targets. Two miRNAs, miR-276-5p and miR-13a-3p, were specifically modulated by RSV in the salivary glands but not in the guts. miR-276-5p was found to enhance RSV secretion from the salivary glands into rice without altering viral load in insects, whereas miR-13a-3p could play a negative role in viral accumulation in insects. Taken together, our findings highlight tissue-specific miRNA responses in vector–virus interactions and uncover distinct roles of salivary gland miRNAs in regulating viral transmission.

**IMPORTANCE:**

Most plant viruses depend on insect vectors for transmission. The salivary glands of insect vectors are the last barrier for these viruses to overcome before being transmitted to plant hosts. In this work, we dissected the microRNA (miRNA) response to plant virus infection in insect salivary glands using the model of the small brown planthopper and rice stripe virus (RSV). The abundance of hundreds of miRNAs changes in the salivary glands after RSV infection. Two specific miRNAs play distinct roles. One enhances the release of RSV from salivary glands into rice plants, and the other regulates viral accumulation within the insects. These findings deepen our understanding of small RNA reactions and potential functions of miRNAs to viral infection in the salivary glands of insect vectors.

## INTRODUCTION

Arboviral diseases account for over 17% of all human infectious diseases and place approximately 80% of the global population at risk (1). More than 70% of plant viruses are transmitted by insects, resulting in annual agricultural losses of up to $60 billion (2). Insects are the primary vectors for arboviruses, which can establish long-term, persistent infections in insect vectors, such as mosquitoes for certain human viruses or planthoppers for rice viruses, often with little to no apparent impact on insects’ physiology (3, 4). Arboviruses typically initiate infection by crossing the midgut epithelium, then spread via the hemolymph to the salivary glands, where they are transmitted to hosts through saliva during feeding or passed vertically via the ovaries (5). The midgut and salivary glands serve as key barriers for viral infection and transmission (6). Unlike the midgut, which supports viral replication, the salivary glands represent the final tissue barrier for cross-kingdom transmission. The ability to cross the salivary gland barrier and the viral load in this tissue are critical for viral transmission.

Traditionally, research on salivary glands has focused on protein effectors, which modulate viral infection by influencing viral persistence, replication, release, and host interactions (7–10). However, the discovery of microRNAs (miRNAs) in saliva and salivary glands of arthropod vectors has revealed a new class of potential regulators in virus–vector interactions (11–14). Unlike proteins that primarily function through protein-protein interactions (8–10), miRNAs exert broad regulatory effects by base-pairing with target mRNAs, influencing gene expression in both vectors and hosts (14–16). Although small RNA sequencing has confirmed the presence of miRNAs in the salivary glands and saliva of mosquitoes, ticks, and planthoppers, their functional roles in viral infection and transmission remain poorly characterized (11–14). Nonetheless, emerging evidence highlights their potential significance. In the small brown planthopper (SBPH), salivary gland-enriched miR-315-5p promotes rice black-streaked dwarf virus infection by suppressing a melatonin receptor in the insect (17). Another SBPH miRNA, miR-263a, enhances rice stripe virus (RSV) replication by directly targeting the 3′ extended terminal region of viral genomic RNA1 and the 5′ UTR of insect *cathepsin B-like* gene (14, 18). In ticks, infection with Powassan virus (POWV) alters salivary miRNA expression, with 35 miRNAs upregulated and 17 downregulated. Functional assays revealed that inhibiting specific upregulated miRNAs—such as isc-miR-315, isc-miR-5307, and several novel candidates—leads to increased POWV levels in host cells, suggesting these miRNAs suppress viral replication (19). Furthermore, bioinformatic analyses indicate that vector salivary miRNAs may target host mRNAs involved in immune and inflammation pathways, potentially shaping the local environment to influence viral infection or secretion (11–13). Notably, one study demonstrated that SBPH saliva delivers miR-263a into rice plants, where it silences *GATA19* to activate jasmonate signaling and enhance plant resistance to RSV—providing direct evidence of salivary miRNA activity in host tissues (14). Despite these advances, the specific role of salivary gland miRNAs in regulating viral secretion during feeding remains poorly understood.

RSV is one of the most destructive rice viruses in East Asia, especially China, Japan, and Korea (20). RSV, a member of the *Tenuivirus* genus, has a genome consisting of four single-stranded RNA segments, encoding a nucleocapsid protein (NP), an RNA-dependent RNA polymerase, and five nonstructural proteins (21). NP gene RNA or NP protein levels are commonly used as reliable indicators of viral load and are standard metrics for detecting and quantifying RSV in both insect vectors and plants (4). RSV is efficiently transmitted between rice plants by SBPH (*Laodelphax striatellus*) in a persistent and circulative-propagative manner (20). The salivary glands play a crucial role in viral transmission, with proteins, such as Importin α2, LssaMP, and Exportin 6, identified as key viral receptors and effectors facilitating viral infection and secretion (7, 8, 22). However, the response of salivary gland miRNAs to RSV infection and their functional significance has not yet been systematically investigated.

In this study, we constructed and compared small RNA libraries from the salivary glands of nonviruliferous and viruliferous SBPH to characterize the overall miRNA response to RSV infection. By analyzing differentially expressed miRNAs and their potential targets, we revealed distinct regulatory patterns for upregulated and downregulated miRNAs. We further investigated two miRNAs specifically modulated by RSV in the salivary glands for their roles in viral accumulation and release.

## RESULTS

### Remodeling of miRNA expression by RSV infection in the salivary glands of small brown planthoppers

**Fig 1:**
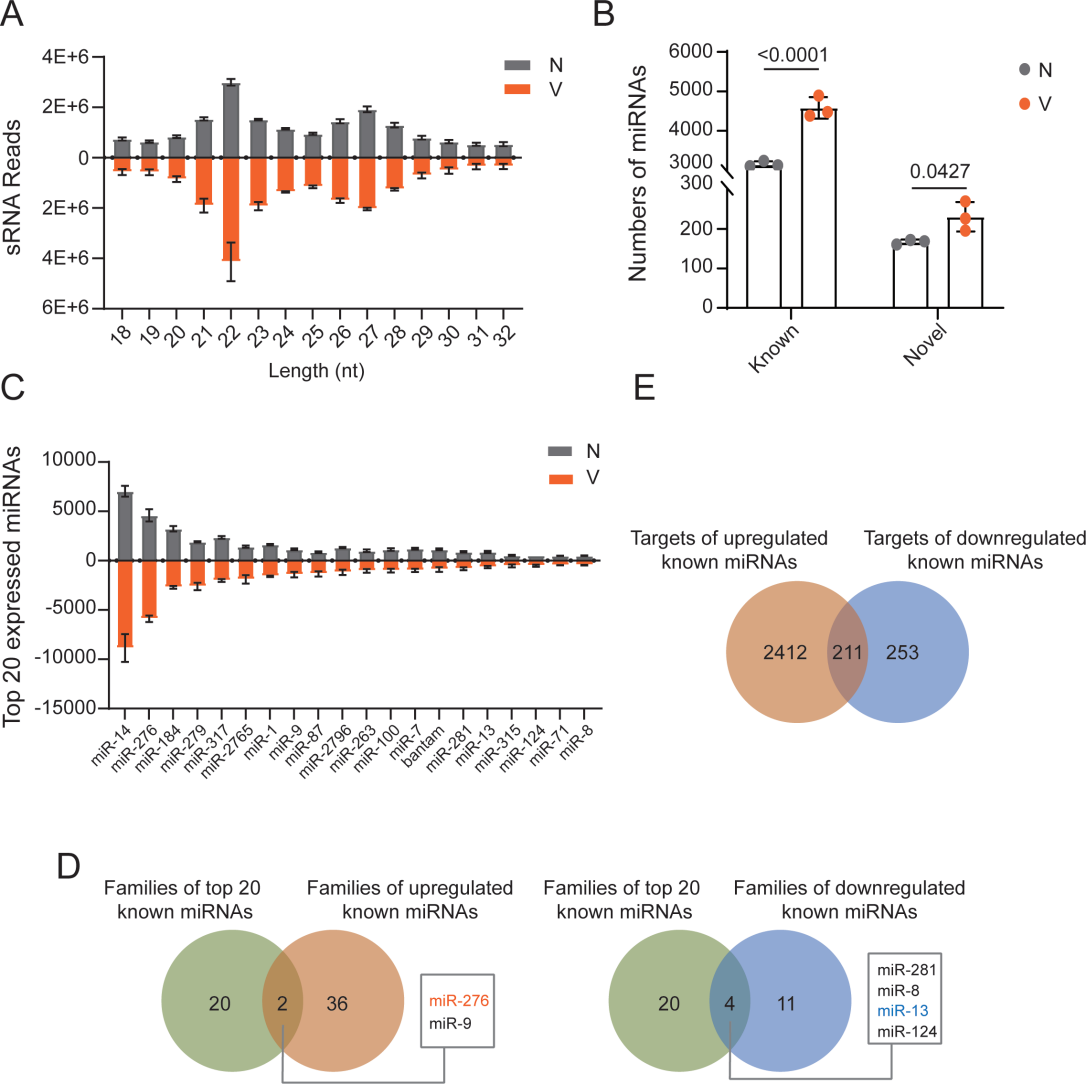
MicroRNA profiling in the salivary glands of nonviruliferous (N) and viruliferous (V) SBPH. (**A**) Size distribution of small RNAs in salivary glands from N and V SBPH. (**B**) Numbers of known and novel miRNAs identified in N and V salivary glands. Data are shown as mean ± SE; statistical significance was assessed by two-tailed Student’s *t*-test (*P* < 0.05). *P* values are indicated on each bar chart. (**C**) The top 20 most abundant miRNAs in the salivary glands of N and V SBPH. (**D**) Venn diagram showing the overlap between miRNA families among the top 20 highly expressed miRNAs and those differentially expressed (upregulated or downregulated) upon RSV infection. Shared miRNA families are listed in the box to the right. Red and blue highlight miRNAs identified in this study that regulate RSV secretion or accumulation. (**E**) Venn diagram illustrating the overlap between predicted target genes of upregulated and downregulated miRNAs.

To elucidate the profiles of miRNAs in the salivary glands of the SBPH during RSV infection, sRNA libraries were constructed and sequenced from salivary gland samples of nonviruliferous (N) and viruliferous (V) fourth-instar nymphs. Three biological replicates for each group were sequenced. Principal component analysis revealed clear separation between N and V samples along principal component 1. Clustering was tighter among N samples, suggesting greater consistency in the uninfected state (Fig. S1). Following the exclusion of adapter sequences and low-quality reads, an average of 21,366,618 and 21,627,593 clean reads were obtained for N and V groups (NCBI number PRJNA1307885) (Table 1). The dominant sizes of sRNAs were 22 and 27 nt, and the size distributions of sRNAs were essentially identical for the two groups (Fig. 1A). A total of 6,277 miRNAs were identified, including 5,909 known miRNAs (Table S1) and 368 novel miRNAs (Table S2). A significantly higher number of miRNAs was detected in V samples compared to N samples (Fig. 1B). Based on sequence similarity of their mature forms, the 5,909 known miRNAs were classified into 201 established miRNA families. Among them, 1,186 miRNAs (|log₂FC| > 1, *P*-adjust < 0.05) were differentially expressed miRNAs (DEMs), including 1,143 known miRNAs. Of these, 1,090 miRNAs were upregulated, representing 36 families, while 53 miRNAs were downregulated, belonging to 11 families (Table S3). Comparison of the top 20 most highly expressed known miRNAs with DEMs revealed that miR-276 and miR-9 families were both upregulated and highly abundant, whereas miR-281, miR-8, miR-13, and miR-124 families were among the downregulated miRNAs yet ranked highly in expression (Fig. 1C and D), suggesting that downregulated miRNAs tend to be more abundantly expressed in salivary glands under RSV infection.

**TABLE 1:** Summary statistics of small RNA sequencing data from the salivary glands of nonviruliferous (N) and viruliferous (V) SBPH

Sample	Raw reads	Clean reads	Useful reads (18–32 nt)	Mapped to the SBPH genome	Mapped percent (%)	Mapped to known miRNA	Mapped to novel miRNA
N1	21837565	20665949	16776594	12558354	74.86	3134202	542108
N2	22946431	21920355	17905740	13266766	74.09	2716540	534611
N3	23068166	21513550	17933736	13624859	75.97	3283823	578998
V1	20027998	19512752	17768902	12723049	71.60	3698943	773617
V2	23786402	22918385	21315565	15596510	73.17	5987141	718869
V3	23946491	22451641	19092275	14421382	75.54	2491585	460347

### Potential regulatory networks of miRNA target genes

**Fig 2:**
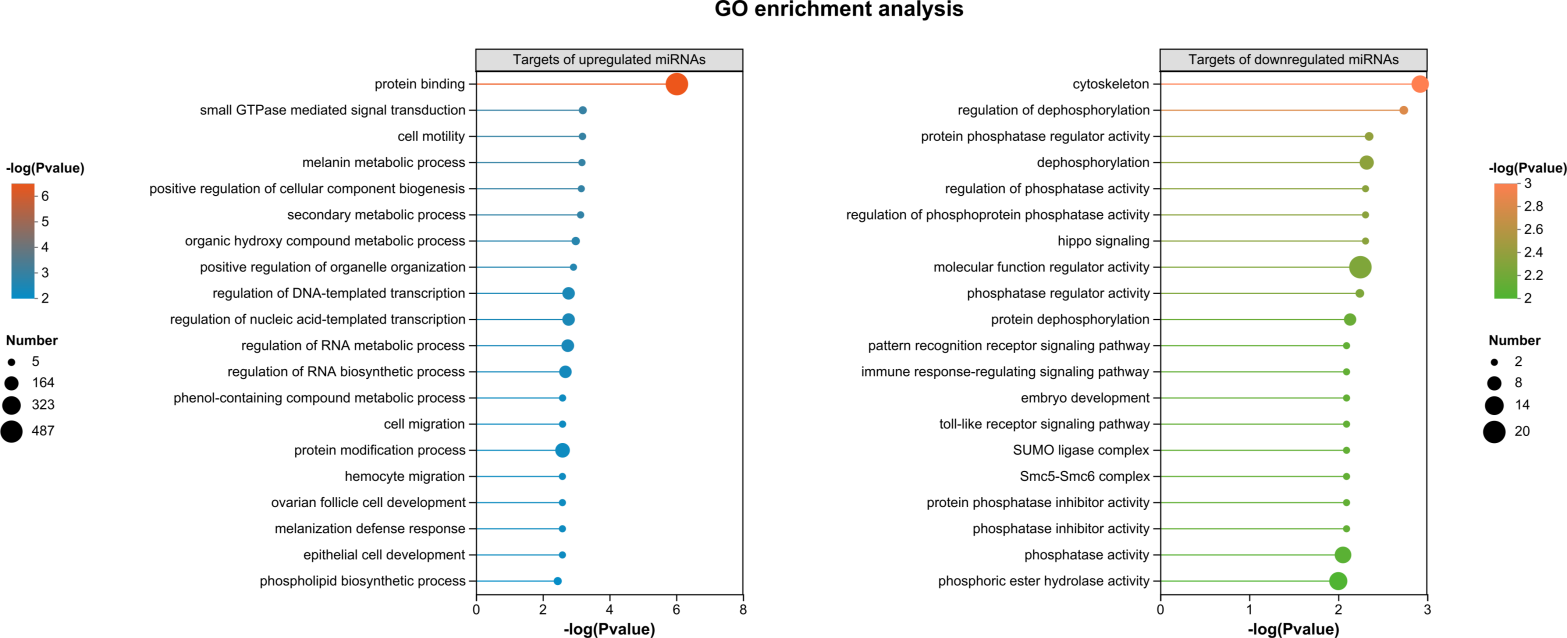
GO enrichment analysis of predicted target genes of known miRNAs differentially expressed in SBPH salivary glands upon RSV infection (*P* value < 0.05).

To investigate the regulatory roles of the known DEMs, target gene prediction was performed, yielding 2,876 targets in total—2,623 for upregulated miRNAs and 464 for downregulated miRNAs, with 211 targets shared between both groups (Fig. 1E). Gene ontology (GO) annotation of these targets revealed that “binding,” “cell part,” and “cellular process” were the most highly represented categories in molecular function, cellular component, and biological process (Fig. S2). In addition, “transcription regulator activity” was specifically obtained in the targets of upregulated miRNAs (Fig. S2A), and “extracellular region” was uniquely associated with the targets of downregulated miRNAs (Fig. S2B). In the GO enrichment analyses (*P* value < 0.05), “protein binding” was the most significantly enriched GO term among the targets of upregulated miRNAs. The remaining 19 enriched GO terms were relatively evenly distributed, with “protein modification process,” “regulation of DNA-templated transcription,” “regulation of nucleic acid-templated transcription,” “regulation of RNA metabolic process,” and “regulation of RNA biosynthetic process” containing the highest numbers of target genes (Fig. 2). For the targets of downregulated miRNAs, the strongest enrichment was observed in “cytoskeleton” and “regulation of dephosphorylation.” The other 18 enriched GO terms were broadly distributed, with “molecular function regulator activity,” “phosphatase activity,” “phosphoric ester hydrolase activity,” and “dephosphorylation” encompassing the largest numbers of target genes (Fig. 2). When *P*-adjust <0.05 was applied for the enrichment analysis, only “protein binding” was significantly enriched for the targets of upregulated miRNAs.

**Fig 3:**
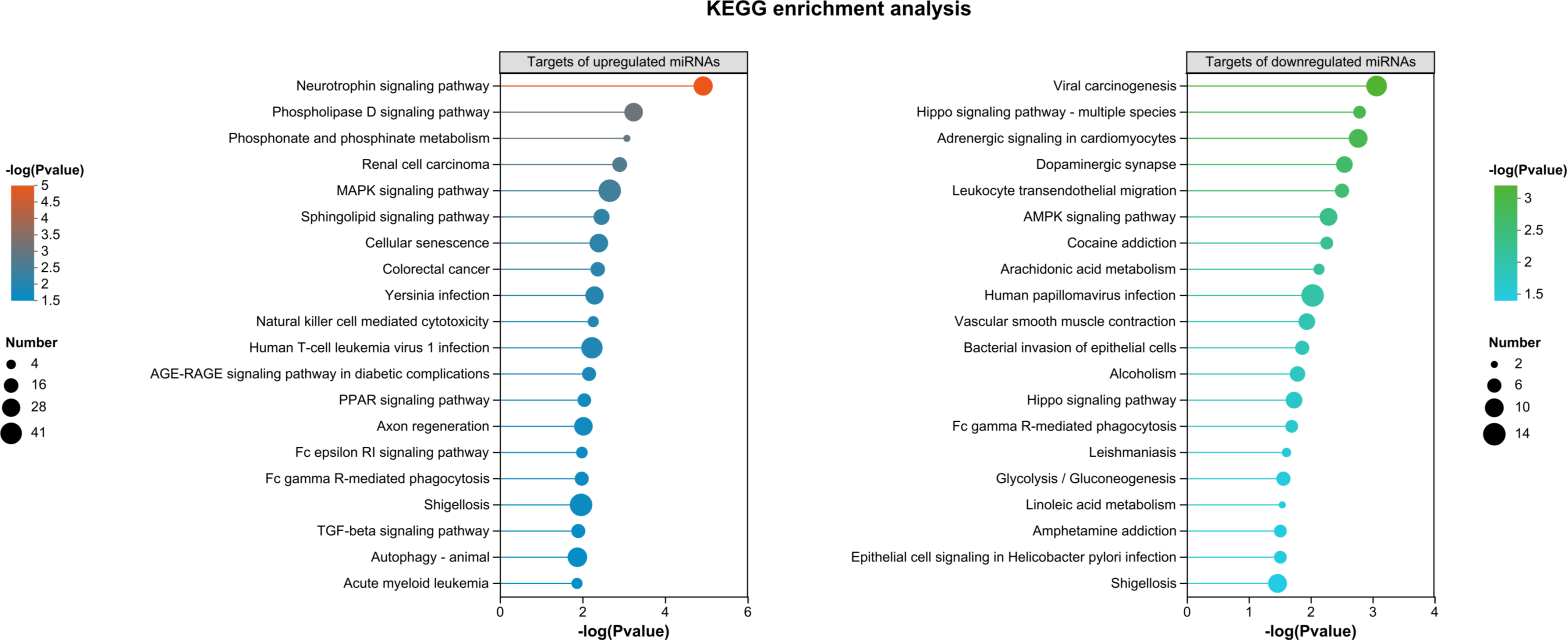
KEGG enrichment analysis of predicted target genes of known miRNAs differentially expressed in SBPH salivary glands upon RSV infection (*P* value < 0.05).

Kyoto Encyclopedia of Genes and Genomes (KEGG) pathway annotation revealed that the targets of upregulated and downregulated miRNAs shared six common pathways, representing the majority of the assigned pathways in both groups (Fig. S3). However, some pathways were unique to each group: folding, sorting, and degradation (under genetic information processing) and lipid metabolism (under metabolism) were specifically to the targets of upregulated miRNAs (Fig. S3A), while circulatory system (under organismal systems) and carbohydrate metabolism (under metabolism) were unique to the targets of downregulated miRNAs (Fig. S3B). In addition, KEGG enrichment analysis (*P* value < 0.05) revealed that the neurotrophin signaling pathway was the most significantly enriched among the targets of upregulated miRNAs, while the remaining 19 enriched pathways were relatively evenly distributed (Fig. 3). In contrast, for the targets of downregulated miRNAs, the 20 enriched KEGG pathways showed a uniform distribution (Fig. 3). When *P*-adjust <0.05 was applied for the enrichment analysis, only the neurotrophin signaling pathway was significantly enriched for the targets of upregulated miRNAs.

### RSV specifically regulates miR-276-5p and miR-13a-3p in salivary glands

**Fig 4:**
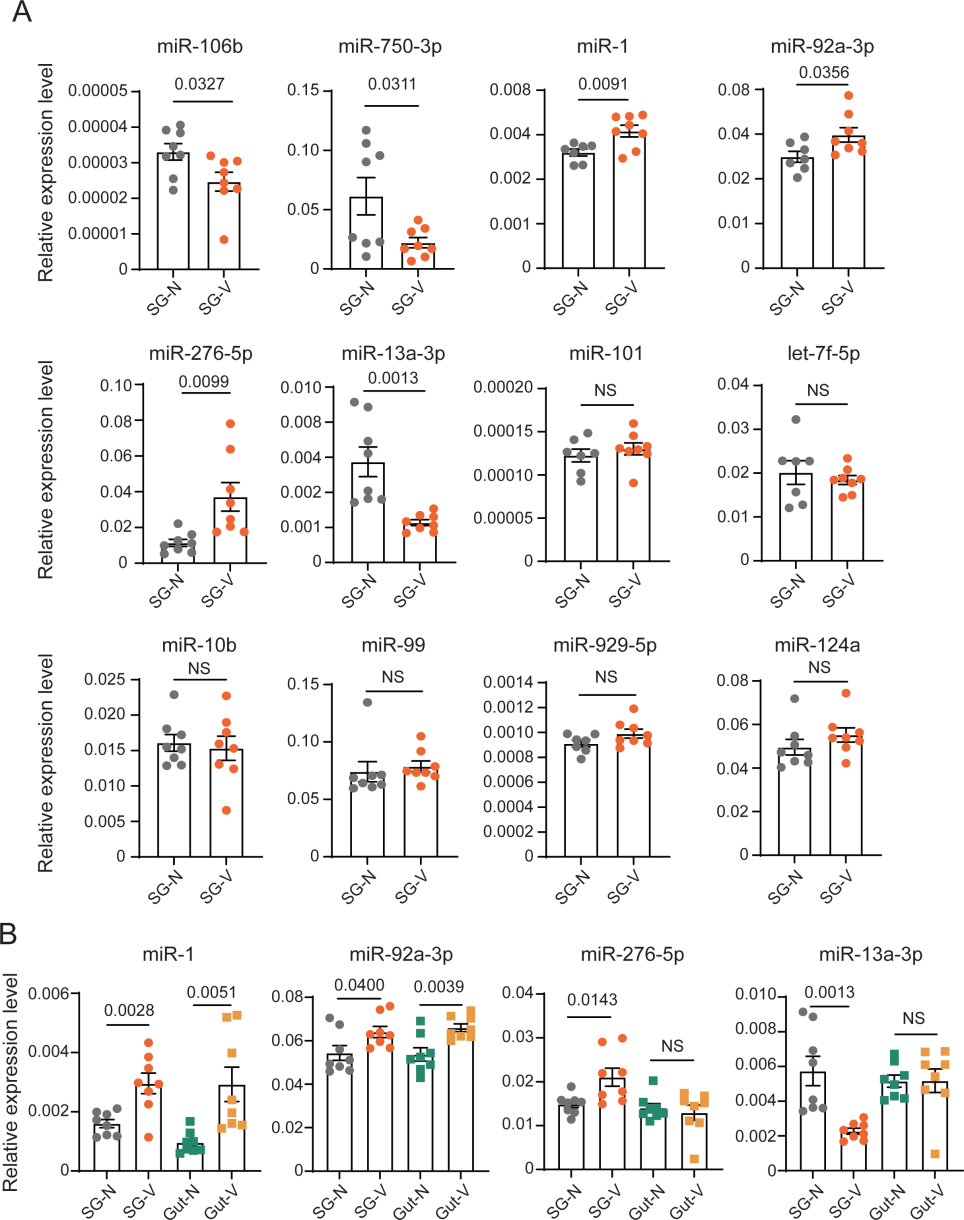
Expression patterns of differentially expressed known miRNAs identified through sRNA-seq. (**A**) Relative expression levels of 12 differentially expressed known miRNAs in the salivary glands of fourth-instar nonviruliferous and viruliferous SBPH nymphs. (**B**) Relative expression levels of four miRNAs (miR-1, miR-92a-3p, miR-276-5p, and miR-13a-3p) in both the salivary glands and guts of fourth-instar nonviruliferous and viruliferous SBPH nymphs. Data are presented as mean ± SE, with statistical significance assessed using a two-tailed Student’s *t*-test (*P* < 0.05). *P* values are indicated on each bar chart; NS, no significant difference.

Among the 1,143 known DEMs, 775 DEMs with an average read count greater than 1 in the V samples, representing 23 miRNA families, were selected for experimental validation. The most abundantly expressed miRNAs from each family were chosen for cloning. Reverse transcriptase PCR (RT-PCR), followed by Sanger sequencing, confirmed the presence of 12 miRNAs, and seven of these showed significant expression changes after RSV acquisition (Fig. 4A; Table S4). Among them, the expression trends of miR-1, miR-92a-3p, miR-276-5p, and miR-13a-3p were consistent with sRNA sequencing results. miR-1, miR-92a-3p, and miR-276-5p were upregulated upon RSV infection, while miR-13a-3p was downregulated.

The expression of the four miRNAs was further compared in the salivary glands and guts with and without RSV infection using quantitative real-time PCR (qPCR). As shown in Fig. 4B, miR-1 and miR-92a-3p were upregulated in both the salivary glands and guts upon RSV infection, suggesting they may function in both tissues during RSV infection. In contrast, miR-276-5p and miR-13a-3p responded to RSV uniquely in the salivary glands but not in the guts. miR-276-5p was specifically upregulated, and miR-13a-3p was specifically downregulated by RSV in the salivary glands. Therefore, we selected these two miRNAs for functional study.

### Effects of miR-276-5p and miR-13a-3p on RSV accumulation and secretion

**Fig 5:**
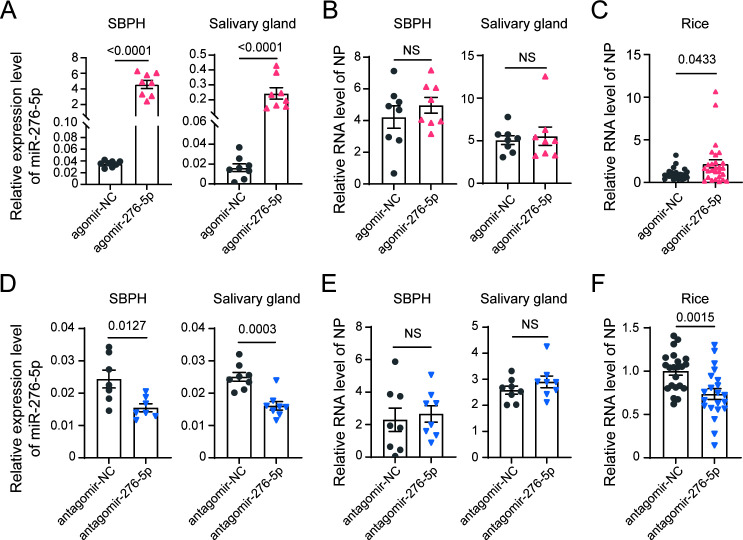
Effects of miR-276-5p on RSV accumulation in SBPH and transmission to rice. (**A–B**) Relative expression of miR-276-5p (**A**) and RSV *NP* RNA levels (**B**) in whole insects and salivary glands of viruliferous fourth-instar SBPH nymphs 4 days after injection with agomir-276-5p or agomir-NC (negative control). (**C**) Relative *NP* RNA levels in rice plants after 1 day of feeding by insects from (**A**). Two independent qPCR experiments were normalized, and *NP* RNA fold changes are relative to the agomir-NC group in panel **C**. (**D–E**) Relative expression of miR-276-5p (**D**) and RSV *NP* RNA levels (**E**) in whole insects and salivary glands of viruliferous SBPH nymphs 4 days after injection with antagomir-276-5p or antagomir-NC. (**F**) Relative *NP* RNA levels in rice after 1 day of feeding by insects from (**D**). Two independent qPCR experiments were normalized, and *NP* RNA fold changes are relative to the antagomir-NC group in panel **D**. Data are shown as mean ± SE; statistical significance was determined by two-tailed Student’s *t*-test (*P* < 0.05). *P* values are indicated on the bar charts; NS, not significant.

To investigate whether miR-276-5p and miR-13a-3p regulate RSV infection, the agomirs (chemically modified double-stranded miRNA mimics) and antagomirs (single-stranded inhibitors) for each miRNA were synthesized and injected into viruliferous SBPH nymphs. The treated insects were allowed to feed on healthy rice seedlings for 1 day. Injection of miR-276-5p agomir significantly increased miR-276-5p amount in the whole body and salivary glands of SBPH (Fig. 5A). No significant changes in viral accumulation in terms of *NP* RNA levels were observed in either whole insects or salivary glands (Fig. 5B). However, rice leaves fed upon by insects treated with miR-276-5p agomir showed significantly higher RNA levels of *NP* compared to those fed upon by agomir-NC-treated insects, indicating the enhanced viral secretion (Fig. 5C). Injection of miR-276-5p antagomir did not affect viral accumulation either (Fig. 5D and E), but miR-276-5p antagomir treatment significantly decreased viral secretion (Fig. 5F). In addition, no significant influence on insect survival was observed after injection with agomir or antagomir of miR-276-5p. These results suggest that miR-276-5p promotes RSV secretion from the salivary glands into host plants without affecting viral replication.

**Fig 6:**
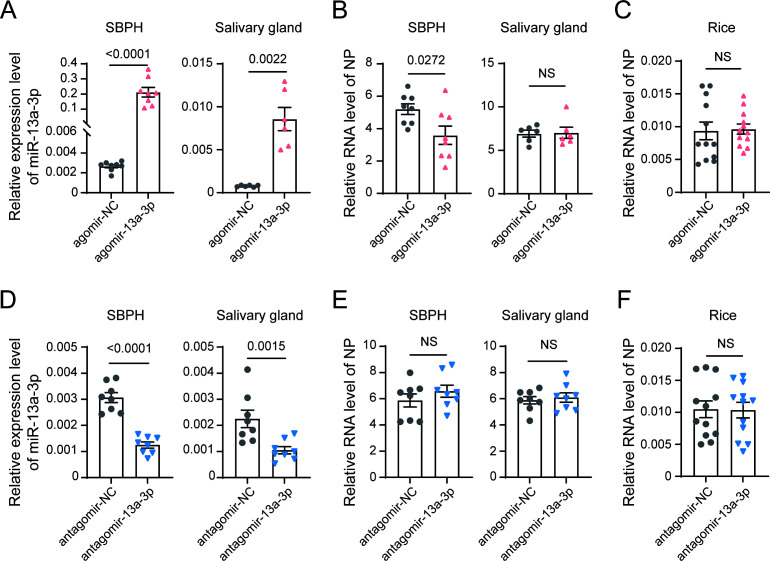
Effects of miR-13a-3p on RSV accumulation in SBPH and transmission to rice. (**A–B**) Relative expression of miR-13a-3p (**A**) and RSV *NP* RNA levels (**B**) in whole insects and salivary glands of viruliferous fourth-instar SBPH nymphs 4 days after injection with agomir-13a-3p or agomir-NC (negative control). (**C**) Relative *NP* RNA levels in rice plants after 1 day of feeding by insects from (**A**). (**D–E**) Relative expression of miR-13a-3p (**D**) and RSV *NP* RNA levels (**E**) in whole insects and salivary glands of viruliferous SBPH nymphs 4 days after injection with antagomir-13a-3p or antagomir-NC. (**F**) Relative *NP* RNA levels in rice after 1 day of feeding by insects from panel **D**. Data are shown as mean ± SE; statistical significance was determined by two-tailed Student’s *t*-test (*P* < 0.05). *P* values are indicated on the bar charts; NS, not significant.

For miR-13a-3p, injection of miR-13a-3p agomir significantly reduced *NP* RNA levels in whole insects but did not affect viral loads in the salivary glands or viral transmission to rice leaves (Fig. 6A through C). miR-13a-3p antagomir treatment only led to a slight but not significant increase in *NP* RNA levels in whole insects with no impact on viral accumulation in the salivary glands (Fig. 6D and E) or virus secretion into rice leaves (Fig. 6F). Additionally, no significant impact on insect survival was observed following injection of either the agomir or antagomir of miR-13a-3p. These findings indicate that miR-13a-3p may play a negative role in viral accumulation in insects.

### Potential regulatory networks of the targets of miR-276-5p

A total of 33 transcripts, corresponding to 30 genes, were predicted as candidate targets of miR-276-5p. Among these, three target genes—*zinc finger protein 2-like*, *guanylate kinase-associated protein Mars*, and *Protein phosphatase 1B*—were predicted by both miRanda and RNAhybrid, lending higher confidence to these interactions; the remaining targets were predicted by only one of the two algorithms (Table S5). GO annotation of these targets revealed that the most highly represented functional categories among these targets were “binding” and “membrane part” (Fig. S4A). Notably, in comparison to the GO profiles of targets for upregulated miRNAs, the “membrane part” category was specifically and prominently represented among miR-276-5p targets, suggesting a specialized role for this miRNA in membrane-associated processes. In the GO enrichment analyses using a *P* value threshold of <0.05, the top three most significantly enriched terms were “N-acetylglucosaminylphosphatidylinositol deacetylase activity,” “RNA polymerase II general transcription initiation factor binding,” and “1-phosphatidylinositol-4-phosphate 3-kinase activity.”. The remaining enriched GO terms were relatively evenly distributed, with “transferase activity, transferring phosphorus-containing groups” encompassing the largest number of target genes (Fig. S4B). However, when the more stringent *P*-adjust <0.05 was applied to correct for multiple testing, no GO terms remained significantly enriched.

KEGG pathway annotation showed that signal transduction (under environmental information processing) was the most frequently represented pathway (Fig. S5A), a trend also observed in the KEGG analysis of targets of both up- and downregulated miRNAs. KEGG enrichment analysis at *P* value <0.05 indicated significant enrichment in pathways, such as FoxO signaling pathway, human immunodeficiency virus 1 infection, oocyte meiosis, and cell cycle (Fig. S5B). Nevertheless, when the *P*-adjust <0.05 criterion was applied, no KEGG pathways were found to be significantly enriched.

In conclusion, the predicted targets of miR-276-5p are enriched in membrane-associated components and key signaling functions—such as 1-phosphatidylinositol-4-phosphate 3-kinase activity and the FoxO signaling pathway—many of which are linked to cytoskeletal organization and membrane trafficking. This suggests a biologically plausible mechanism by which miR-276-5p could influence viral stability or secretion in the salivary glands through the regulation of membrane dynamics and intracellular signaling pathways. However, these enrichments did not reach statistical significance after correction for multiple testing (*P*-adjust <0.05), indicating that the pathway-level evidence remains suggestive rather than conclusive. Therefore, while the predicted regulatory network provides valuable hypotheses for how miR-276-5p may modulate host processes relevant to viral transmission, it should be viewed as a starting point for further investigation. Experimental validation of individual target genes will be essential to fully understand the functional role of miR-276-5p in the virus-insect-host interaction.

## DISCUSSION

Salivary glands are critical determinants of arbovirus transmission. In this study, we present a comprehensive profile of the miRNA response to RSV in the salivary glands of SBPH, revealing distinct regulatory patterns between upregulated and downregulated miRNAs following viral infection. We identified two salivary gland-specific miRNAs that respond to RSV and play divergent roles in viral accumulation and transmission, potentially fine-tuning different stages of the viral transmission cycle. Our findings offer new insights into the molecular interplay between insect vectors and plant arboviruses and highlight promising targets for disrupting the transmission of insect-borne plant viruses.

While both the salivary glands and guts are essential for viral replication and transmission, they often display distinct molecular and functional responses across various vector–virus systems. For example, in *Aedes aegypti* mosquitoes infected with dengue virus, 190 genes showed differential expression in the salivary glands, whereas 13 genes were differentially expressed in the guts. Only the *tubulin beta chain* was differentially regulated in both tissues (23). In *Ae. aegypti* infected with Zika virus, the gut mounts a robust antiviral response, primarily through activation of the siRNA and piRNA pathways, whereas the salivary glands create a more permissive environment, potentially facilitating transmission via immune suppression. There, immune-related pathways, such as Toll, IMD, and JAK/STAT, show more pronounced transcriptional changes (24). Our previous transcriptomic analysis of the SBPH revealed a stronger immune response to RSV in the salivary glands than in the guts. Specifically, RSV infection in the guts upregulates genes related to lysosomal function, digestion, and detoxification, while suppressing those involved in DNA replication and repair. In the salivary glands, RSV promotes RNA transport but suppresses key signaling pathways, such as MAPK, mTOR, Wnt, and TGF-β (25). In this study, we characterized the miRNA response to RSV infection in the salivary glands and identified potential differences in regulatory mechanisms between the two tissues. We found that miR-1 and miR-92a-3p are responsive to RSV infection in both tissues, whereas miR-276-5p and miR-13a-3p are specifically regulated in the salivary glands upon viral infection. This suggests that miR-1 and miR-92a-3p may indirectly affect viral accumulation or secretion in the salivary glands by modulating viral replication in the guts. In contrast, miR-276-5p and miR-13a-3p likely play direct roles in regulating viral stability or secretion within the salivary glands, making them promising targets for strategies aimed at blocking the horizontal transmission of RSV. These findings highlight the tissue-specific nature of host–virus interactions and underscore the unique role of the salivary glands in modulating the balance between viral persistence and transmission.

Emerging studies have uncovered the dynamics of salivary miRNAs in vector-borne virus transmission. For example, sRNA-seq analysis of *Ae. aegypti* and *Aedes albopictus* saliva identified 103 exogenous miRNAs. Notably, 59 and 30 known miRNAs were upregulated in the saliva of chikungunya virus (CHIKV)-infected *Ae. aegypti* and *Ae. albopictus*, respectively, suggesting these miRNAs may play functional roles in CHIKV dissemination and transmission (11). However, a more recent study comparing miRNA expression in both saliva and salivary glands in non-infected and CHIKV-infected *Ae. aegypti* only identified two upregulated and three downregulated miRNAs in saliva, while no significant changes in miRNAs were observed in the salivary glands. Instead, CHIKV infection primarily activated the siRNA and piRNA pathways (26). In female *Ixodes scapularis* ticks infected with POWV, sRNA sequencing revealed 379 salivary gland miRNAs with 35 upregulated and 17 downregulated. Functional assays showed that inhibiting specific upregulated miRNAs, such as isc-miR-315, isc-miR-5307, and several novel candidates, increased viral levels in host cells, indicating these miRNAs act to suppress viral replication (19). In our study, RSV infection upregulated 1,090 miRNAs and downregulated 53 miRNAs in the salivary glands of SBPH. These results collectively indicate that miRNA response to arboviruses may be tissue- and context-specific and highlight the complexity of vector–virus interactions across different systems. Investigating miRNA dynamics in the salivary glands would offer valuable insights into the complex interactions between viruses and their insect vectors.

We found that miR-276-5p enhanced RSV transmission by promoting viral secretion into rice plants. miR-276 is a highly conserved miRNA family and has been reported in many insects, including *Ae. aegypti*, *Drosophila melanogaster*, *Bactrocera dorsalis*, *Tribolium castaneum*, *Locusta migratoria*, and *Aphis gossypii* (27). This miRNA family consists of two mature forms—miR-276-5p and miR-276-3p. They are primarily known to regulate key biological processes, such as insect development, metabolism, and reproduction. For instance, in *D. melanogaster*, miR-276-3p suppresses the insulin signaling pathway by repressing *InR* expression, ultimately resulting in decreased developmental growth (28). In *L. migratoria*, miR-276-3p promotes the egg-hatching synchrony by upregulating *brm* expression in the ovaries (27). In *Anopheles coluzzii*, blood meal-induced miR-276-5p fine-tunes the expression of *branched-chain amino acid transferase*, contributing to the termination of the reproductive cycle (29). Additionally, in *A. gossypii*, miR-276-5p enhances susceptibility to the insecticide spirotetramat by downregulating *acetyl-CoA carboxylase* in resistant strains (30). We demonstrated that miR-276-5p enhances RSV transmission by facilitating viral secretion into rice plants without affecting viral load within insects. Further studies are required to explore the potential mechanisms for this specific phenomenon in the future.

## MATERIALS AND METHODS

### Insect preparation

Nonviruliferous (N) and viruliferous (V) SBPH populations were maintained separately on rice seedlings (*Oryza sativa* subsp. *japonica*) Wuyujing 3 in glass incubators at 24°C under a 16 h light/8 h dark photoperiod. The RSV-carrying rates of the viruliferous SBPH populations were monitored every 3 months using a dot enzyme-linked immunosorbent assay with homemade monoclonal anti-NP antibodies (4).

### RNA extraction and sRNA‐seq library construction

Total RNA was separately extracted from the salivary glands of nonviruliferous and viruliferous fourth-instar SBPH nymphs. For each condition, three biological replicates were prepared, with 30 salivary glands per replicate. RNA extraction was performed using TRIzol reagent (Invitrogen, Carlsbad, CA, USA; 15596026) following the manufacturer’s protocol. The quality and concentration of each RNA sample were assessed by 1.2% agarose gel electrophoresis and by measuring the OD260/OD280 ratio using a NanoDrop One spectrophotometer (Thermo Scientific, Waltham, MA, USA; 840-317400). One microgram of prepared total RNA with a concentration ≥50 ng/µL, RQN >7, OD260/280 between 1.8 and 2.2 was ligated to the 5′ and 3′ adaptors and reverse transcribed into cDNAs using the QIAseq miRNA Library Kit (Qiagen, Germany). The adapter-ligated cDNA was enriched by PCR, then purified and size-selected on a 6% Novex TBE PAGE gel to isolate small RNA fragments of 18–30 nt. Final libraries were sequenced using 140–160 bp single-end reads on the NovaSeq X Plus platform at Majorbio Bio-Pharm Technology Co., Ltd., Shanghai, China.

### Analysis of sRNA sequencing data

sRNA sequencing data were analyzed using the online platform of Majorbio Cloud Platform. Briefly, clean reads mapped to the SBPH reference transcript database (31) were aligned against miRBase (32) and Rfam (33) databases to annotate known miRNAs and other non-coding RNAs. Unannotated reads were analyzed using miRDeep2 to predict novel miRNAs (34). Expression levels of known and novel miRNAs were quantified and normalized using transcripts per million. DEMs between N and V samples were identified using DESeq2 with the following thresholds: a minimum twofold change in expression (log₂ fold change >1 or <–1) and an adjusted *P* value <0.05 (35).

### Target prediction and functional enrichment analysis

Target genes of known DEMs were predicted using miRanda (36) and RNAhybrid (37) algorithms on the Majorbio Cloud Platform, based on the SBPH genome databases (31). Prediction relied on the “seed region” rule, requiring base pairing between positions 2–8 of the mature miRNA and the target mRNA. For miRanda, the parameters were set as follows: score cutoff ≥160, energy cutoff ≤−18 kcal/mol, and strict mode enabled. For RNAhybrid, the energy cutoff was set to ≤−18 kcal/mol and the *P* value cutoff to ≤0.05. A gene was considered a candidate target if predicted by at least one of the two algorithms.

Predicted target genes were functionally annotated using public databases, including GO, KEGG, EggNOG, NR, Swiss-Prot, Pfam, and Rfam. GO enrichment analysis was performed using the Python package Goatools (https://github.com/tanghaibao/GOatools), with significance defined by both *P* value and adjusted *P* value <0.05. KEGG pathway enrichment was conducted in R using Fisher’s exact test, and pathways with a *P* value and adjusted *P* value <0.05 were considered statistically enriched.

### cDNA synthesis and quantitative PCR

Total RNA was extracted from 10 salivary glands, eight guts, five whole insect bodies of nonviruliferous and viruliferous fourth-instar SBPH nymphs, or two rice leaves using TRIzol reagent (Invitrogen). For miRNA quantification, 2 µg of total RNA was reverse transcribed into cDNA using the miRcute Plus miRNA First-Strand cDNA Synthesis Kit (Tiangen, Beijing, China; KR211) according to the manufacturer’s instructions (14). For viral RNA detection, 2 µg of RNA was reverse transcribed using the SuperScript III First-Strand Synthesis System (Thermo Scientific; 18080051) with random hexamer primers (Promega, Madison, WI; C1181).

qPCR was performed on a LightCycler 480 Instrument II (Roche, Basel, Switzerland). miRNA expression levels were measured using the miRcute miRNA qPCR Detection Kit (Tiangen; FP411), while mRNA and viral RNA transcripts were amplified using LightCycler 480 SYBR Green I Master (Roche; 04887352001). The qPCR reaction system was a 20 µL volume, consisting of 10 µL of 2× SYBR Green I Master Mix (Roche) or 2× miRcute miRNA premix (Tiangen), 4 µL of cDNA template, and 0.5 µL of each primer (10 µM). For the evaluation of miRNA expression, the forward primer for each target miRNA was designed based on its mature sequence, while the reverse primer was provided by the miRcute miRNA qPCR Detection Kit (Tiangen). All qPCR products were sequenced to confirm primer specificity. In SBPH, *EF2* (Contig0.299) was used as the reference gene for normalizing viral *NP* levels, and U6 snRNA was used as the endogenous control for miRNA expression (18). In rice, *UBQ10* (LOC4328390) served as the internal control for normalization of viral *NP* level (14). Each experiment included six to eight biological replicates for insect samples and at least 12 replicates for rice samples. All primer sequences are provided in Table S6.

### Injection of miRNA agomir or antagomir

Agomirs and antagomirs targeting miR-276-5p and miR-13a-3p were designed and synthesized by GenePharma (Shanghai, China). Agomirs, which function as miRNA mimics, were delivered as chemically modified dsRNAs with the following sequences: for miR-276-5p (sense: 5′-AGCGAGGUAUAGAGUUCCUACG-3′, antisense: 5′-UAGGAACUCUAUACCUCGCUUU-3′), for miR-13a-3p (sense: 5′-UAUCACAGCCACUUUGAUGUGGU-3′, antisense: 5′-CACAUCAAAGUGGCUGUGAUAUU-3′), and a negative control (NC) agomir (sense: 5′-UUCUCCGAACGUGUCACGUTT-3′, antisense: 5′-ACGUGACACGUUCGGAGAATT-3′). Antagomirs, designed to inhibit endogenous miRNA function, were chemically modified ssRNAs complementary to the mature miRNA sequences: miR-276-5p (5′-CGUAGGAACUCUAUACCUCGCU-3′), miR-13a-3p (5′-ACCACAUCAAAGUGGCUGUGAUA-3′), with a scrambled sequence (5′-CAGUACUUUUGUGUAGUACAA-3′) used as the NC antagomir. A volume of 13.8 nL of each oligonucleotide (250 µM) was microinjected into viruliferous fourth-instar SBPH nymphs using a Nanoliter 2000 microinjection system (World Precision Instruments). Four days after injection, whole insects or dissected salivary glands were collected for RNA extraction to assess the impact of miRNA upregulation or inhibition on RSV accumulation.

### Determination of RSV secretion by salivary glands

To assess the effect of miRNA modulation on RSV secretion, groups of 15 viruliferous fourth-instar SBPH nymphs were microinjected with agomir, antagomir, or their respective NC. After 3 days of incubation to allow for miRNA modulation, the treated insects were transferred onto 3-week-old healthy rice seedlings (15 insects per plant) and allowed to feed for 1 day. The insects were then carefully removed, and the rice leaves were collected for viral detection. Two leaves from each plant were used as one biological replicate, and at least 12 replicates were prepared per treatment group. RSV accumulation in the rice leaves was quantified by qPCR to determine the relative RNA level of *NP*.

### Statistical analysis

All bar charts were created using GraphPad Prism 9 (GraphPad Software, San Diego, CA) based on original experimental data. Statistical significance between two groups was assessed using Student’s *t-*test.

## Data Availability

All data are present in the paper and the supplemental material. The sRNA data have been deposited in the NCBI database with the accession number PRJNA1307885.

## SUPPLEMENTAL MATERIAL

Supplemental material jvi.01434-25-s0001.pdf Figures S1 to S5; Table S6.

Table S1 jvi.01434-25-s0002.xlsx List of 5,909 known miRNAs identified from small RNA sequencing of salivary glands in nonviruliferous and viruliferous small brown planthoppers.

Table S2 jvi.01434-25-s0003.xlsx List of 368 novel miRNAs identified from small RNA sequencing of salivary glands in nonviruliferous and viruliferous small brown planthoppers.

Table S3 jvi.01434-25-s0004.xlsx Differentially expressed miRNAs identified between nonviruliferous and viruliferous salivary glands using DESeq2 with a threshold of {greater than or equal to}2-fold change and adjusted *P*-value < 0.05.

Table S4 jvi.01434-25-s0005.xlsx List of 23 known miRNAs selected for experimental validation by reverse transcription PCR (RT-PCR) followed by Sanger sequencing.

Table S5 jvi.01434-25-s0006.xlsx Predicted candidate targets of miR-276-5p by using RNAhybrid and miRanda.

ASM does not own the copyrights to Supplemental Material that may be linked to, or accessed through, an article. The authors have granted ASM a non-exclusive, world-wide license to publish the Supplemental Material files. Please contact the corresponding author directly for reuse.
