# Supplementary material for: MicroRNA response in insect salivary glands to plant virus infection
Source: J Virol. 2025 Oct 29;99(11):e01434-25. doi: 10.1128/jvi.01434-25 (PMC12646003; doi:10.1128/jvi.01434-25)
Supplement: Supplemental material — Figures S1 to S5; Table S6. [file jvi.01434-25-s0001.pdf]

Supplemental Material

MicroRNA response in insect salivary glands to plant virus infection

Yan Xiao[1,2], Guohua Liang[1], Jiaming Zhu[1,2], Feng Cui[1,2*], Wan Zhao[1,2*]

[1]State Key Laboratory of Animal Biodiversity Conservation and Integrated Pest Management, Institute of Zoology, Chinese Academy of Sciences, Beijing 100101, China

[2]University of Chinese Academy of Sciences, Beijing 100049, China

*Authors for correspondence:

Feng Cui

Tel: +86-10-64807218, Email: cuif@ioz.ac.cn.

Wan Zhao

Tel: +86-10-64806299, Email: zhaow@ioz.ac.cn.

This PDF includes Figure S1 to S5, and Table S6. Tables S1 to S5 are provided as separate Excel files.

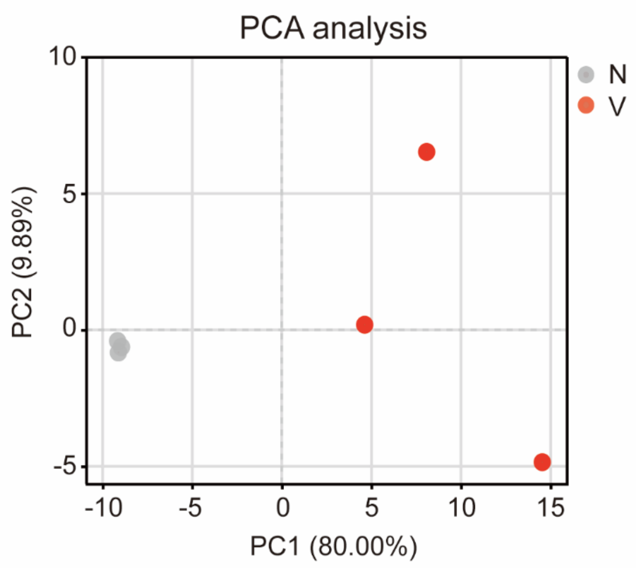

Fig. S1. Principal component analysis (PCA) of salivary gland samples from nonviruliferous (N) and viruliferous (V) small brown planthopper (SBPH).

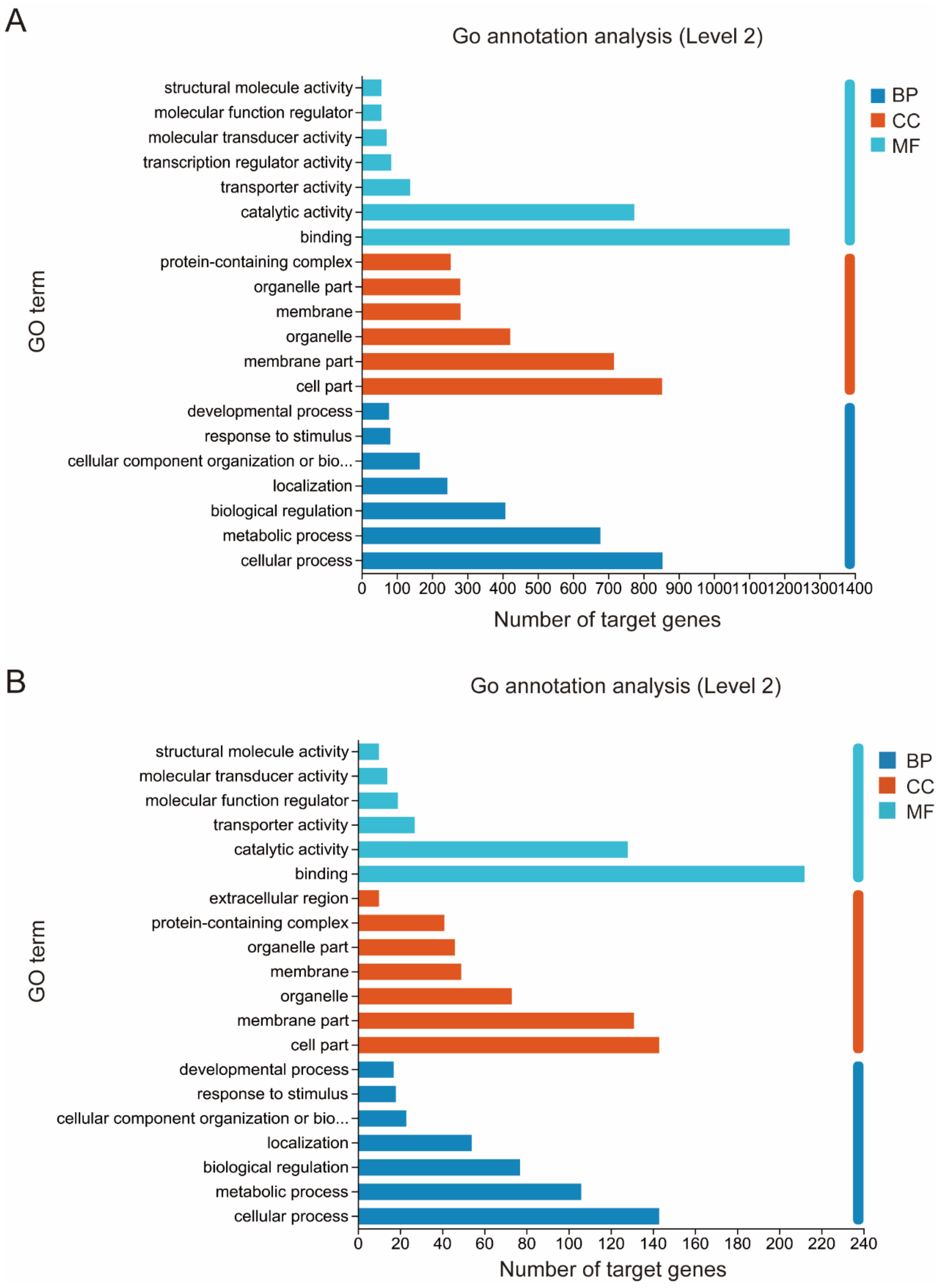

Fig. S2. GO annotation analysis of predicted target genes of known miRNAs differentially expressed in SBPH salivary glands upon RSV infection. (A) Upregulated miRNAs; (B) Downregulated miRNAs. The top 20 GO terms are listed based on the number of associated genes. BP, Molecular Function; CC, Cellular Component; MF, Molecular Function.

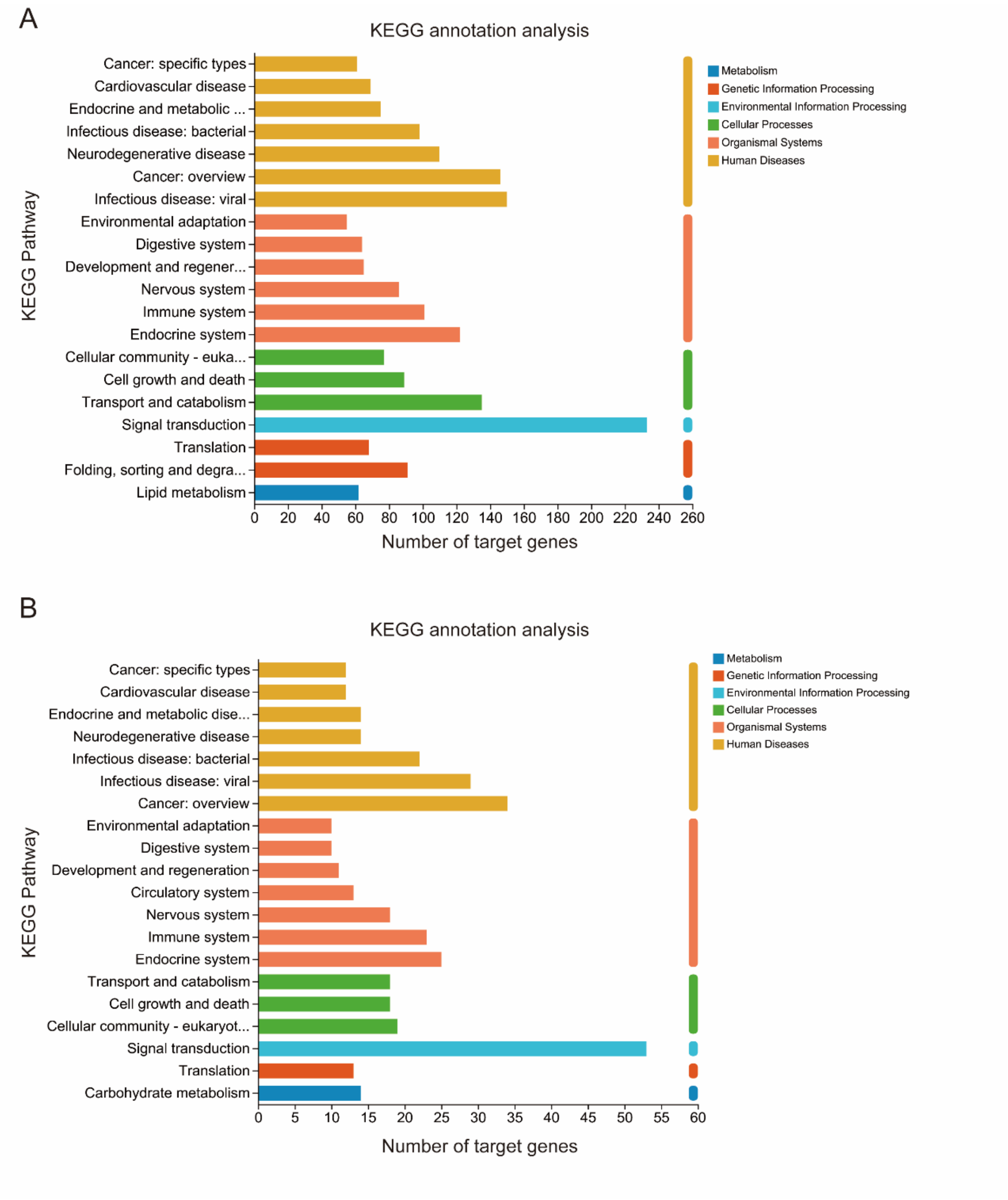

Fig. S3. KEGG pathway annotation of predicted target genes of known miRNAs differentially expressed in SBPH salivary glands upon RSV infection. (A) Upregulated miRNAs; (B) Downregulated miRNAs. The top 20 KEGG pathways are listed based on the number of associated genes.

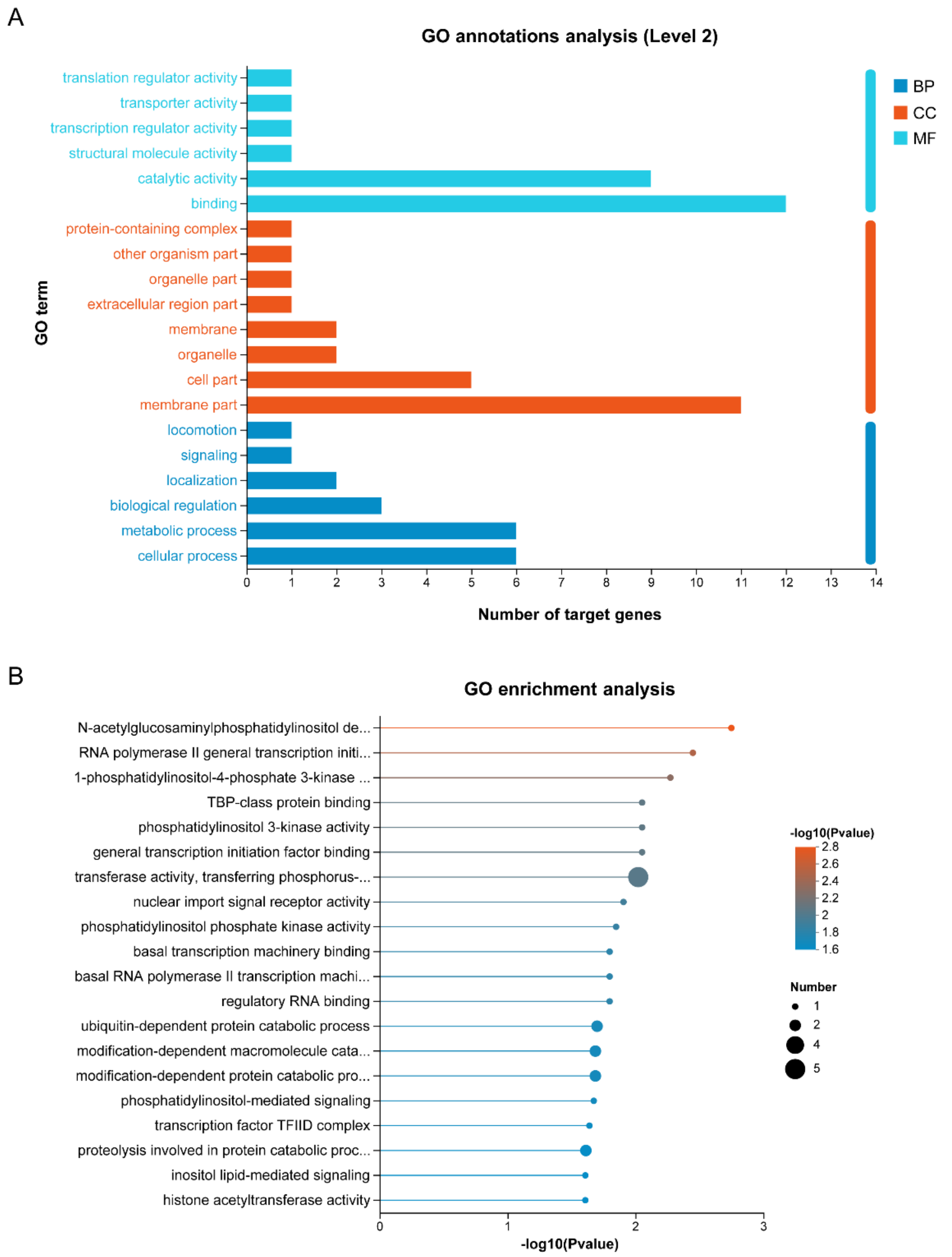

Fig. S4. GO annotation and enrichment analysis of predicted target genes of miR-276-5p. (A) GO annotation at level 2 for predicted target genes of miR-276-5p. BP, Molecular Function; CC, Cellular Component; MF, Molecular Function; (B) GO enrichment analysis of predicted target genes of miR-276-5p (p-value < 0.05).

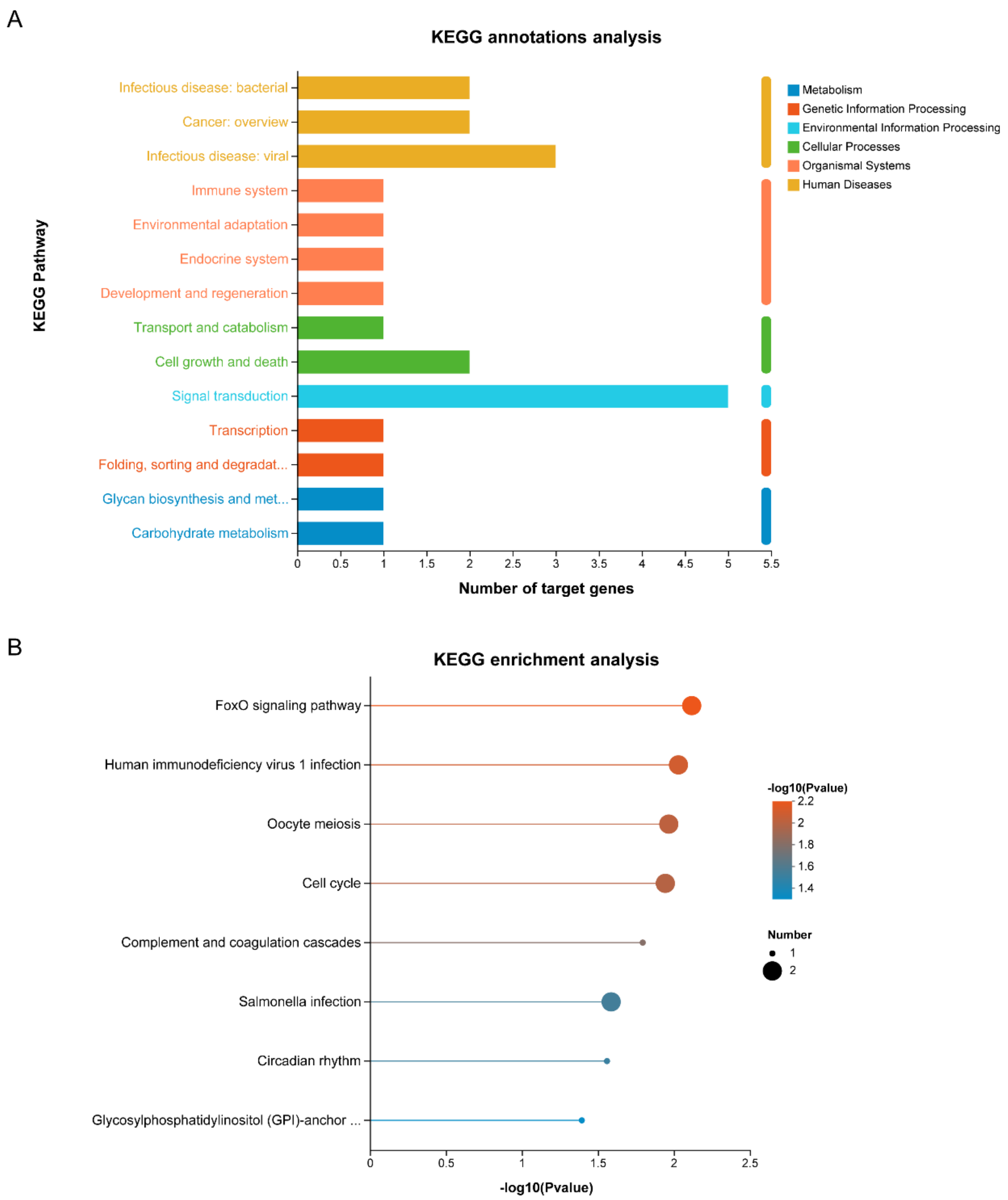

Fig. S5. KEGG annotation and enrichment analysis of predicted target genes of miR-276-5p. (A) KEGG annotation at level 2 of predicted target genes of miR-276-5p; (B) KEGG enrichment analysis of predicted target genes of miR-276-5p (p-value < 0.05).

Table S1. (separate file)

List of 5, 909 known miRNAs identified from small RNA sequencing of salivary glands in nonviruliferous and viruliferous small brown planthoppers.

Table S2. (separate file)

List of 368 novel miRNAs identified from small RNA sequencing of salivary glands in nonviruliferous and viruliferous small brown planthoppers.

Table S3. (separate file)

Differentially expressed miRNAs identified between nonviruliferous and viruliferous salivary glands using DESeq2 with a threshold of ≥2-fold change and adjusted *p*-value < 0.05.

Table S4. (separate file)

List of 23 known miRNAs selected for experimental validation by reverse transcription PCR (RT-PCR) followed by Sanger sequencing.

Table S5. (separate file)

Predicted candidate targets of miR-276-5p by using RNAhybrid and miRanda.

Table S6. Primers used in this study.

Primer name	Sequence (5’ to 3’)
EF2-q-F	GTCTCCACGGATGGGCT
EF2-q-R	ATCTTGAATTTCTCGGCATACAT
UBQ10-q-F	TCACCTACGTCTACAACCAG
UBQ10-q-R	AGTGCTGATCGTATTGACAGA
NP-q-F	GGAACAAATGCCAATGCTATC
NP-q-R	TGAGACATTTGGGAATAGCTGA
U6-q-F	TGGAACGATACAGAGAAGATTAGCA
U6-q-R	AACGCTTCACGATTTTGCGT
miR-106b-F	TAAAGTGCTGACAGTGCAGATAA
miR-750-3p-F	CCAGATCTAACTCTTCCAGCTC
miR-1-F	TGGAATGTAAAGAAGTATGTATA
miR-92a-3p-F	GTATTGCACTTGTCCCGGCCTGTA
miR-276-5p-F	AGCGAGGTATAGAGTTCCTACGAA
miR-13a-3p-F	TATCACAGCCACTTTGATGTGGTAAA
miR-101-F	CGTACAGTACTGTGATAACTGAAAA
Let-7f-5p-F	GTGAGGTAGTAGATTGTATAGTTA
miR-10b-F	TACCCTGTAGAACCGAATTTGTGAAA
miR-99-F	CAACCCGTAGATCCGATCTTGTGAA
miR-929-5p-F	GAAATTGACTCTAGTAGGGAGTCAAA
miR-124a-F	TAAGGCACGCGGTGAATGCC
miR-26a-5p-F	GTTCAAGTAATCCAGGATAGGCTAA
miR-103-3p-F	GAGCAGCATTGTACAGGGCTATGAAA
miR-21-5p-F	TAGCTTATCAGACTGATGTTGACAA
miR-215-5p-F	ATGACCTATGATTTGACAGACAA
miR-126-3p-F	TCGTACCGTGAGTAATAATGCGAA
miR-16a-F	TAGCAGCACGTAAATATTGGCGAA
miR-143-F	TGAGATGAAGCACTGTAGCTCAA
miR-183-5p-F	GTATGGCACTGGTAGAATTCACTAA

miR-27b-3p-F	TTCACAGTGGCTAAGTTCTGCAAA
miR-46-3p-F	GCTGTCATGGAGTTGCTCTCTTCAA
miR-281-3p-F	GCTGTCATGGAGTTGCTCTCTTTGAA

F, forward primers; R, reverse primers.
